# Cross‐Sectional and Longitudinal Comparison of Commonly Used Screening Tools for Bipolar Disorders

**DOI:** 10.1111/bdi.70133

**Published:** 2026-06-21

**Authors:** Anna Tröger, Yiqi Zeng, Thomas Richardson, Emma Palmer‐Cooper, Allan H. Young, Rebecca Strawbridge

**Affiliations:** ^1^ Department of Psychological Medicine, Institute of Psychiatry, Psychology & Neuroscience King's College London London UK; ^2^ Department of Psychiatry and Psychotherapy University Hospital Carl Gustav Carus, Technische Universität Dresden Dresden Germany; ^3^ Southampton Psychosis and Bipolar Research and Innovation Group, School of Psychology University of Southampton Southampton UK

**Keywords:** bipolar disorder, diagnosis, HCL‐32, MDQ, psychometric properties, screening tools

## Abstract

**Background:**

Misdiagnosis is common in bipolar disorder (BD). Currently used screening tools are brief and cost‐effective, but there is a lack of understanding of their reliability over time, as well as whether responses are influenced by demographic or clinical factors.

**Aims:**

To examine the cross‐sectional and longitudinal reliability and validity of two commonly used screening tools, as well as their associations with current mood states and other participant characteristics.

**Methods:**

A total of 331 adult patients with a diagnosis of BD completed the Mood Disorder Questionnaire (MDQ) and the Hypomania Symptom Checklist‐32 (HCL‐32) as well as measures of mood online at baseline and three months later.

**Results:**

The MDQ was found to have low reliability with poor internal consistency (α = 0.531; α = 0.647 at respective timepoints) and test–retest reliability (*r*_*s*_ = 0.582). The HCL‐32 was more reliable with good internal consistency (α = 0.815 at both timepoints) and test–retest reliability (*r*_*s*_ = 0.725). MDQ scores were significantly positively associated with comorbidities and current symptoms of activation/mania, and negatively correlated with age and years since last hospitalisation. The HCL‐32 was also significantly positively correlated with activation/mania, and negatively correlated with age, years since diagnosis and years since last hospitalisation. BD‐I patients scored higher than other BD subtypes. Mood state was the only variable with a significant change over time.

**Conclusions:**

The HCL‐32 appeared more reliable than the MDQ, although all participants needed to exceed an MDQ threshold at baseline to participate in the study, which may have biased our results.

## 1 Introduction

Bipolar disorder (BD) is a chronic affective disorder characterised by (hypo‐)manic and depressive episodes, interspersed with euthymic episodes. BD is often undiagnosed [1] or misdiagnosed [2], in part due to the complexity of symptoms, episodes and comorbidities [3]. Distinguishing BD from major depressive disorder (MDD) is particularly challenging because the index episode in BD is frequently depression, while first (hypo‐)manic symptoms can appear years later, and depressive symptoms may account for up to 80% of time spent in episodes [4, 5]. Consequently, the delay between symptom onset and correct diagnosis averages around ten years [6] often resulting in treatment discordant with BD management guidelines. This is problematic as inappropriate treatments may worsen BD trajectories, can lead to severe adverse consequences, such as antidepressant‐induced switches to mania [7] or increased suicide risk [8] and is unlikely to change unless the DSM‐5 or ICD‐11 criteria are revised.

Thus, correct and timely BD diagnosis is essential. Various self‐report measures that allow for a brief and cost‐effective BD screening have been developed. The Mood Disorder Questionnaire [9] (MDQ) and the Hypomania Symptom Checklist‐32 [10] (HCL‐32) are two of the most widely used screening tools for BD [11] that have been developed to indicate a potential differentiation of BD from MDD in research and practice. Both measures have been validated in many countries, but the psychometric properties and the comparability of the two measures in head‐to‐head comparisons have been inconsistent [12, 13, 14, 15, 16]. The only meta‐analysis on studies directly comparing the psychometric properties of the MDQ and the HCL‐32 found acceptable psychometrics for both instruments and comparable accuracy of both tools, with higher specificity of the MDQ [17]. However, none of the studies included in the meta‐analysis examined the comparability of the test–retest reliability of the two measures. It is important to consider long‐term reliability and validity in BD screening, as recall bias can have a significant impact on the retrospective report of earlier (hypo)manic episodes [14]. Indeed, to our knowledge, the only study assessing longitudinal psychometric properties of the MDQ found that while the validity for detecting a recent (hypo)manic episode was excellent with optimal sensitivity and specificity, it was poor with limited accuracy of recalling manic symptoms after two years [14].

The present study aimed to compare the psychometric properties of the MDQ and HCL‐32 in a sample of participants with BD, both cross‐sectionally and between two assessments three months apart. Specifically, the objectives of this study were:

1. To examine the reliability of both screening tools, measuring their internal consistency and test–retest reliability over a three‐month period.

2. To examine the validity of both screening tools, indicated by a rate of false negative occurrences (i.e., participants with BD not meeting screening thresholds at one or both timepoints).

3. To explore whether current mood states, as well as other clinical and sociodemographic factors, are associated with screening tool scores cross‐sectionally.

4. To explore whether clinical characteristics explain changes in screening scores over time.

Based on previous findings and the somewhat short follow‐up period of three months, we hypothesised that both the MDQ and the HCL‐32 are reliable with adequate internal consistency (Cronbach's alpha > 0.7) and test–retest reliability (*r* > 0.7). Additionally, we expected both measures to be valid instruments in the detection of BD, with less than 10% of participants not meeting thresholds for BD on both screening tools. We did not specify hypotheses for the latter two objectives, in light of scant and inconsistent evidence to date [18, 19, 20].

## 2 Methods

### 2.1 Participants

Participants were included if they declared having a formal diagnosis of BD from a healthcare professional, met the MDQ threshold for BD (score of ≥ 7), were at least 18 years old and were fluent in English. Participants with a cyclothymia diagnosis were excluded, as both the MDQ and HCL‐32 are not designed to reliably screen for cyclothymia.

### 2.2 Procedure

This current data set is a secondary analysis of data collection from a study looking at psychological mechanisms and how these impacted mood symptoms over time in Bipolar Disorder [21]. Participants were recruited online via study adverts on social media, including Twitter, Facebook, Instagram and TikTok, and through online support groups and charities associated with BD (Bipolar UK, Crest BD). Several patient‐report online questionnaires measuring demographics, affective symptoms, and quality of life, as well as the two bipolar screening tools, were sent through an automatically generated email at two time points (time 1: baseline and time 2: follow‐up) three months apart between August 2021 and January 2022. Online informed consent was obtained from all participants prior to study participation. Their participation was voluntary, and they were free to discontinue their participation at any time during the study. Participants were entered into a prize draw to win one of twelve gift vouchers upon completion. All study procedures were approved by the University of Southampton Ethics Committee (ERGO II ID: 64021).

### 2.3 Measures

#### 2.3.1 Demographic and Clinical Background Information

Information was collected pertaining to age, gender (male, female, other (non‐binary or prefer not to say)), ethnicity (white, non‐white), BD type (BD‐I, BD‐II, other BD), years since diagnosis, previous hospitalisation for BD (yes, no; and if yes, years since last hospitalisation), diagnosed by (GP/family doctor, psychiatrist, psychologist, or other mental health service practitioner), other mental health diagnosis (yes, no), type of comorbidity (anxiety disorder, other comorbidity, both anxiety and other comorbidity), currently in mental health services (yes, no), currently under medication (yes, no), and neurodiversity (yes, no).

#### 2.3.2 Symptom Questionnaires

The Internal State Scale (ISS) [22] was used to examine patient‐reported affective symptoms over the last 24 h. The ISS provides subscale scores for depression index (DI), perceived conflict (PC), activation (ACT), wellbeing (WB), as well as a single item representing global severity. It is a 16‐item measure, with items such as “Today I feel impulsive” and “Today I feel depressed”, with each item ranging from 0 (not at all or rarely) to 100 (very much so or much of the time). A systematic review confirmed its good psychometric properties and clinical utility [23].

The Altman Self‐Rating Mania Scale (ASRM) [24] was used as a measure of patient‐reported manic symptoms over the prior week. It includes five questions, which are rated from 0 (not present) to 4 (present to a severe degree), e.g., ratings for the sleep disturbance item range from “I do not need less sleep than usual” to “I can go all day and night without any sleep and still not feel tired”. This measure has good reported psychometric properties and clinical utility [23].

The Center for Epidemiologic Studies Depression Scale (CES‐D) [25] is a 20‐item measure of patient‐reported depressive symptoms over the prior week, such as “I felt hopeful about the future” and “I felt that people disliked me”, with each item ranging from 0 (rarely or none of the time) to 3 (most or all of the time). Meta‐analyses have established its high diagnostic accuracy [26].

The Recovering Quality of Life (ReQoL‐10) [27, 28] questionnaire was used to indicate patient‐reported quality of life, with ten items such as “I felt happy” and “I felt unable to cope” ranging from 0 (none of the time) to 4 (most or all of the time). It has been found to have good psychometric properties in different samples [29, 30].

#### 2.3.3 Screening Tools for BD

The MDQ is a self‐administered screening tool for BD consisting of three parts: (1) 13 yes/no questions assessing (hypo)manic symptoms at any point in their life when they ‘were not [their] usual self’, (2) one yes/no question about the coexistence of these symptoms and (3) one 4‐point scale measuring how much of a problem these symptoms were causing (from no problem to serious problem) [9]. Higher scores indicate a higher probability for BD, with a cut‐off score of 7 usually being considered a positive screen, which was also adopted in this study.

The HCL‐32 is a self‐report screening measure to identify hypomanic features in patients with MDD to recognise BD [10]. Responders are asked to remember a “period when they were in a ‘high’ state” and indicate in 32 yes/no questions the presence of hypomanic symptoms in emotions, behaviours and thoughts during those periods. In eight further questions, the severity of the symptoms' impact is assessed. A cut‐off of 14 “yes” replies is usually used to identify BD and was the threshold used in this study.

### 2.4 Data Analysis

All statistical analyses were completed with SPSS 29.0 for Windows. Firstly, descriptive statistics across variables were calculated. ISS scores were divided into six variables: Depression index, perceived conflict, activation, well‐being, overall severity and mood state [mania or hypomania, mixed state, euthymia, depression] [31]. As recommended, for both screening tools, only the items within the first sections were used in scoring (see measures [9, 10]).

#### 2.4.1 Objective 1: Reliability Examination

To measure the reliability of each screening tool, we assessed the internal consistency of the MDQ and HCL‐32, using Cronbach's alpha coefficients at both timepoints (baseline and follow‐up three months later), respectively. A Cronbach's alpha > 0.7 was considered good internal consistency [32].

To measure the impact of time on the screening tool scores, we analysed the test–retest reliability with Spearman's rank correlation coefficient between both time‐points for both screening tools (MDQ and HCL‐32), respectively. A correlation of > 0.7 was considered good test–retest reliability [32].

#### 2.4.2 Objective 2: Validity Examination

The proportion (%) of participants who did not meet the thresholds for BD on each screening tool was calculated at each time point separately. Non‐statistically, we examined the demographic characteristics of all participants who failed to meet scoring thresholds on either measure at either time point.

#### 2.4.3 Objective 3: Associations of BD Screening Scores With Current Mood States, Clinical and Sociodemographic Factors

We examined the association between MDQ and HCL‐32 scores with current mood states, clinical, and sociodemographic factors, cross‐sectionally. We used Spearman's rank correlation analyses for all continuous variables (ISS, ASRM, CES‐D, ReQoL, years since diagnosis, age at diagnosis), Mann–Whitney U Tests for binary variables (ethnicity, medication status, psychiatric comorbidity, lifetime hospitalisation), and Kruskal–Wallis tests for polychotomous variables (gender, diagnosed bipolar subtype). If a Kruskal–Wallis test was significant, we performed post hoc Dunn's tests and reported which groups were significantly (*p* < 0.05) different from each other.

#### 2.4.4 Objectives 4: Associations With Clinical Characteristics Changes

Paired samples *t*‐tests were conducted to compare the two scores between both time points for each questionnaire measuring symptoms (ISS continuous variables, ASRM, CES‐D, ReQoL). Chi‐Squared test was used to compare the mood states variable from the ISS (see above) between the two time‐points.

Two multiple linear regressions were conducted to examine the relationship between all clinical and sociodemographic characteristics that were found to be significantly associated with the respective screening tool in objective 3 (independent variables) and changes in the MDQ and HCL‐32 screening scores, respectively (respective BD screening score at follow‐up as dependent variable, respective screening score at baseline as covariate).

## 3 Results

### 3.1 Demographic Characteristics of the Sample

A total of 331 people with BD aged between 18 and 79 years old (*M* = 46, SD = 14.2) participated. Most of the sample was female (*n* = 233, 70%) and white (*n* = 306, 92%). Most participants had a BD‐II diagnosis (*n* = 135, 41%), while 27% (*n* = 90) were diagnosed with BD‐I. All participants had received a formal diagnosis, mainly from a psychiatrist (*n* = 308, 93%). Most people were undergoing treatment for BD during the study, with 59% (*n =* 194) being in mental health services and 92% (*n =* 306) taking psychotropic medication. Detailed characteristics of the participants are described in Table 1.

**TABLE 1:** Demographic and clinical characteristics of the sample.

Demographic characteristics	Participants (*n* = 331)
Gender (*n*, %)	
Male	91 (27.5)
Female	233 (70.4)
Other	7 (2.1)
Ethnicity White (*n*, %)	306 (92.4)
Age (M, SD)	46 (14.2)
Type of BD (*n*, %)	
BD‐I	90 (27.2)
BD‐II	135 (40.8)
Other BD	106 (32.0)
Diagnosed by (*n*, %)	
GP/family doctor	8 (2.4)
Psychiatrist	308 (93.1)
Psychologist	7 (2.1)
Other mental health service practitioner	8 (2.4)
Years since diagnosis (M, SD)	9 (8.8)
With other mental health diagnoses (*n*, %)	125 (37.8)
Type of comorbidity (*n*, %)	
Anxiety comorbidity	35 (10.6)
Other comorbidity	60 (18.1)
Both anxiety and other comorbidities	26 (7.9)
Currently within mental health service (*n*, %)	194 (58.6)
Currently under medication (*n*, %)	306 (92.4)
Previously been hospitalised for BD (*n*, %)	196 (59.2)
Years since last hospitalisation (M, SD)	9 (8)
Neurodiversity (*n*, %)	83 (25.1)

*Note:* Due to missing values, sample sizes, numbers, and percentages do not always add up to 100%.

Abbreviations: BD‐I, bipolar disorder type I; BD‐II, bipolar disorder type II; GP, general practitioner; M, mean; SD, standard deviation.

### 3.2 Descriptive Statistics of the Screening Tools

Descriptive statistics of screening tool scores at both time points are presented in Table 2.

**TABLE 2:** Descriptive statistics of MDQ and HCL‐32.

Screening tool	Statistics	Time 1	Time 2
MDQ	*N* *M (SD)*	331 11.813 (1.387)	194 11.634 (1.637)
	Range	7–13	4–13
	Participants who met the threshold (%)	100^a^	99
HCL‐32	*N* *M (SD)*	330 25.574 (4.312)	172 25.187 (4.383)
	Range	7–32	5–32
	Participants who met the threshold (%)	98.5	98.8

Abbreviations: HCL‐32, Hypomania Checklist‐32; M, mean; MDQ, Mood Disorder Questionnaire; SD, standard deviation.

^a^ As per the inclusion criteria for the study.

### 3.3 Objective 1: Reliability Examination

The MDQ was not indicated to have good internal consistency, with Cronbach's alpha below 0.7 at both timepoints (α = 0.531 at time 1, α = 0.647 at time 2). For the HCL‐32, Cronbach's alpha coefficients indicated good internal consistency (α = 0.815 at both time 1 and 2). The MDQ also did not show good test–retest reliability, with a Spearman's rank correlation between both timepoints below 0.7 (*r*_*s*_ = 0.582). The HCL‐32 displayed acceptable test–retest reliability (*r*_*s*_ = 0.725).

### 3.4 Objective 2: Validity Examination

Less than 10% participants did not meet the threshold for BD on either screening tool at either time point (score of ≥ 7 for the MDQ, ≥ 14 for the HCL‐32; see Table 2). As per the inclusion criteria for the study, all participants met the MDQ threshold at time 1. At time 2, agreement between the MDQ scores and psychiatric diagnosis was high at 99%. For the HCL‐32, agreement was also high at both timepoints (98.5% and 98.8%, respectively). Nine individuals failed to meet the threshold of the MDQ at time 2, or the HCL‐32 at time 1 or time 2. None of these did not meet the threshold on both screening tools or at both timepoints, although follow‐up data were missing for four of those not meeting thresholds at baseline. The characteristics of these nine individuals appeared somewhat similar to the sample as a whole, with age ranging from 20 to 75, and a preponderance of female gender (89%). All of these participants reported having obtained their diagnosis from a psychiatrist, and most were taking medication for BD during the study (89%). However, there appeared to be fewer participants who were white (78%, compared to 92% in the whole sample) and within mental health services (33%, compared to 59% in the whole sample).

### 3.5 Objective 3: Associations of Screening Scores With Clinical and Demographic Factors

Table 3 summarises the cross‐sectional associations between each screening tool at each time point with all clinical and sociodemographic factors, as well as mood states. We found statistically significant positive correlations between MDQ scores and activation (ISS; *r*_*s*_ = 0.159, *p* = 0.004), perceived conflict (ISS; *r*_*s*_ = 0.117, *p* = 0.033), and manic symptoms (ASRM; *r*_*s*_ = 0.254, *p* < 0.001) as well as a negative correlation with age (*r*_*s*_ = −0.119, *p* = 0.939) and years since last hospitalisation (*r*_*s*_ = −0.142, *p* = 0.047) at time 1. Moreover, participants with comorbid mental illnesses showed MDQ score differences from those without comorbid psychiatric diagnoses (*U* = 10522.5, *Z* = 2.892, *p* = 0.004), with participants with comorbidities scoring higher (mean rank of 184.58) than those without (mean rank of 154.73). Kruskal–Wallis analyses indicated significant differences among different ISS mood states in MDQ scores at time 1 (*χ2* = 14.015, *p* = 0.003). Patients in (hypo)manic mood states scored significantly higher (mean rank of 198.22) than patients who were depressed (mean rank of 155.05) or euthymic (mean rank of 151.07), but not higher than patients in mixed states (mean rank of 182.17).

**TABLE 3:** Associations of BD screening scores with current mood states, clinical and sociodemographic factors.

Time	Variable	MDQ	*p*‐value	HCL‐32	*p*
		Test statistic		Test statistic	
1	ISS Global	*r*_*s*_ = 0.099	0.073	*r*_*s*_ = 0.127*	0.021
	ISS Activation	*r*_*s*_ = 0.159**	0.004	*r*_*s*_ = 0.117*	0.034
	ISS Wellbeing	*r*_*s*_ = 0.070	0.205	*r*_*s*_ = 0.140*	0.011
	ISS Perceived conflict	*r*_*s*_ = 0.117*	0.033	*r*_*s*_ = 0.069	0.208
	ISS Depression index	*r*_*s*_ = 0.013	0.813	*r*_*s*_ = −0.099	0.071
	ASRM	*r*_*s*_ = 0.254***	< 0.001	*r*_*s*_ = 0.211***	< 0.001
	ReQoL	*r*_*s*_ = −0.010	0.852	*r*_*s*_ = 0.070	0.211
	CESD	*r*_*s*_ = 0.068	0.216	*r*_*s*_ = −0.012	0.834
	Age	*r*_*s*_ = −0.119*	0.030	*r*_*s*_ = −0.192***	< 0.001
	Years since diagnosis	*r*_*s*_ = −0.040	0.468	*r*_*s*_ = −0.137*	0.013
	Years since last hospitalisation	*r*_*s*_ = −0.142*	0.047	*r*_*s*_ = −0.219**	0.002
	Ethnicity (white/non‐white)	*U* = 3483.0; *Z* = 0.781	0.435	*U* = 3181.5; *Z* = −1.381	0.167
	Currently in MH service (yes/no)	*U* = 12087.5; *Z* = −1.473	0.141	*U* = 12262.0; *Z* = −1.126	0.260
	Currently under medication (yes/no)	*U* = 3775.5; *Z* = −0.113	0.910	*U* = 3609.5; *Z* = −0.444	0.657
	Previously been hospitalised for BD (yes/no)	*U* = 13154.0; *Z* = −0.093	0.926	*U* = 12977.5; *Z* = −0.218	0.828
	With other mental health diagnoses (yes/no)	*U* = 10522.5; *Z* = −2.892**	0.004	*U* = 11451.5; *Z* = −1.624	0.104
	Neurodiversity category (yes/no)	*U* = 9811.5; *Z* = −0.669	0.503	*U* = 9150.0; *Z* = −1.468	0.142
	Gender (male/female/other)	*χ2 = 0*.228	0.892	*χ2 =* 0.665	0.717
	BD type (BD‐I/BD‐II/Other BD)	*χ2 =* 4.636	0.098	*χ2 =* 7.221*	0.027
	Diagnosed by^a^	*χ2 =* 2.465	0.482	*χ2 =* 3.073	0.380
	Type of comorbidity^b^	*χ2 =* 1.638	0.441	*χ2 =* 2.92	0.232
	ISS Mood states^c^	*χ2 =* 14.015**	0.003	*χ2 =* 7.057	0.070
2	ISS Global	*r*_*s*_ = 0.067	0.387	*r*_*s*_ = 0.113	0.141
	ISS Activation	*r*_*s*_ = 0.115	0.136	*r*_*s*_ = 0.109	0.153
	ISS Wellbeing	*r*_*s*_ = 0.057	0.462	*r*_*s*_ = 0.082	0.288
	ISS Perceived conflict	*r*_*s*_ = 0.124	0.105	*r*_*s*_ = 0.114	0.138
	ISS Depression index	*r*_*s*_ = −0.009	0.904	*r*_*s*_ = 0.006	0.937
	ASRM	*r*_*s*_ = 0.111	0.149	*r*_*s*_ = 0.025	0.748
	ReQoL	*r*_*s*_ = −0.048	0.535	*r*_*s*_ = −0.022	0.774
	CESD	*r*_*s*_ = 0.070	0.361	*r*_*s*_ = 0.113	0.139
	ISS Mood states	*χ2 =* 2.231	0.526	*χ2 =* 6.973	0.073

*Note:* ISS mood states include mania or hypomania, mixed state, euthymia, and depression.

Abbreviations: ASRM, Altman Self‐Rating Mania Scale; BD‐I, bipolar disorder type I; BD‐II, bipolar disorder type II; CES‐D, Center for Epidemiologic Studies Depression Scale; GP, general practitioner; HCL‐32, Hypomania Checklist‐32; ISS, Internal State Scale; MDQ, Mood Disorder Questionnaire; ReQoL, Recovering Quality of Life.

* *p* < 0.05.

** *p* < 0.01.

*** *p* < 0.001.

^a^ Diagnosed by (GP or family doctor/psychiatrist/psychologist/other mental health service practitioner).

^b^ Type of comorbidity (anxiety comorbidity/other comorbidity/both anxiety and other comorbidity).

^c^ ISS Mood state (mania or hypomania/mixed state/euthymia/depression).

HCL scores were positively correlated with global severity (ISS; *r*_*s*_ = 0.127, *p* = 0.021), activation (ISS; *r*_*s*_ = 0.117, *p* = 0.034), wellbeing (ISS; *r*_*s*_ = 0.140, *p* = 0.011), and manic symptoms (ASRM; *r*_*s*_ = 0.211, *p* < 0.001), and were negatively correlated with age (*r*_*s*_ = −0.192, *p* < 0.001), years since diagnosis (*r*_*s*_ = −0.137, *p* = 0.013), and years since last hospitalisation (*r*_*s*_ = −0.219, *p* = 0.002) at time 1. Significant differences between patients with different types of BD in the HCL‐32 scores at time 1 were also found (*χ2* = 7.221, *p* = 0.027), with BD‐I patients having higher HCL‐32 scores (mean rank of 188.46) than BD‐II (mean rank of 157.2) and other BD diagnoses (mean rank of 156.47), with a significant difference between the BD‐I and the BD‐II group. No significant associations at time 2 were identified.

### 3.6 Objective 4: Associations With Clinical Characteristics Changes

We examined the changes of all scales measuring clinical characteristics between the two timepoints (supplement S1) and the associations between screening tool score changes and variables which were significantly correlated with them in objective 3. The ISS mood state category was the only variable to significantly change over time (*χ2* = 61.854, *p* < 0.001), and the changes in screening tool scores were not associated with clinical characteristics (Table 4).

**TABLE 4:** Associations between screening score changes and baseline clinical characteristics.

Outcome variable	Predictor variable	Unstandardised B	*p*	95% CI Lower Bound	95% CI Upper Bound
MDQ change^a^	ISS Activation	0.001	0.705	−0.006	0.009
	ISS Perceived conflict	0.000	0.862	−0.004	0.005
	ISS Mood states	0.175	0.551	−0.415	0.764
	ASRM	−0.056	0.273	−0.158	0.046
	Age	0.004	0.836	−0.034	0.041
	Years since last hospitalisation	−0.033	0.408	−0.113	0.047
	With other mental health diagnoses (yes/no)	−0.225	0.454	−0.829	0.379
HCL‐32 change^a^	ISS Global	−0.003	0.916	−0.051	0.046
	ISS Activation	−0.002	0.631	−0.010	0.006
	ISS Wellbeing	−0.001	0.868	−0.014	0.012
	ASRM	0.022	0.827	−0.176	0.219
	Age	−0.044	0.167	−0.107	0.019
	Years since diagnosis	−0.024	0.587	−0.113	0.065
	Years since last hospitalisation	0.007	0.888	−0.091	0.105
	Type of bipolar disorder^b^	0.169	0.681	−0.646	0.984

*Note:* ISS mood states include mania or hypomania, mixed state, euthymia, and depression.

Abbreviations: ASRM: Altman Self‐Rating Mania Scale; CI: confidence interval; HCL‐32: Hypomania symptom checklist‐32; ISS Mood state (mania or hypomania/mixed state/euthymia/depression); ISS: Internal State Scale; MDQ: Mood Disorder Questionnaire.

^a^ Time 2 adjusted for time 1.

^b^ Type of bipolar disorder (BD‐I/BD‐II/Other BD).

## 4 Discussion

This study aimed to compare the psychometric properties of the MDQ and HCL‐32 in BD participants both cross‐sectionally and over a three‐month period. To the best of our knowledge, this is the first paper to review their psychometric properties in a head‐to‐head longitudinal as well as cross‐sectional fashion. Specifically, we examined their internal consistency, test–retest reliability, validity, and associations with current mood states and other clinical and sociodemographic factors, as well as whether these characteristics can explain changes in screening scores over time. Contrary to our first hypothesis, we found that the MDQ's internal consistency and test–retest reliability were poor while the HCL‐32 had adequate internal consistency and test–retest reliability. In line with our second hypothesis, the high proportion of participants meeting BD thresholds on both screening tools indicated good validity. Additionally, we found significant correlations between screening scores and several clinical and sociodemographic factors. At baseline, higher scores on both measures were associated with increased symptoms of mania, younger age, and less time since the most recent hospitalisation. Higher MDQ scores were also associated with having more comorbidities, while higher HCL scores were found in those with a greater overall symptom severity score, in those with a more recent bipolar diagnosis and BD type I (vs. type II/NOS). Screening scores did not appear to change over time, and changes were not related to clinical factors.

### 4.1 Reliability Examination

In contrast to our findings, most previous studies have found good reliability for the MDQ. A possible explanation could be that we were unable to validate the participants' diagnoses, potentially resulting in false positives in our sample. For example, there is a large and complex symptom overlap and comorbidity between BD and borderline personality disorder (BPD) [33], so it is possible that patients with BPD were misdiagnosed as BD, which could have contributed to our differing results. Moreover, Boschloo [14] has also reported the potential limited reliability of the MDQ over the longer‐term (2 years). The authors argue that recall bias might influence retrospective ratings of past manic symptoms, which might also play a role in our sample, despite a significantly shorter time span. As for the HCL‐32, our results are consistent with previous studies suggesting good reliability cross‐sectionally [17] as well as over a three‐month period. Thus, when comparing these two screening tools, our results suggest that the HCL‐32 might be more reliable and stable than the MDQ.

### 4.2 Validity Examination

As expected, we found high agreement between meeting the threshold for BD on the screening tools and formal BD diagnoses. Agreements were even higher than in previous studies [13, 15], which may be attributable to our inclusion criteria for participants: As we excluded BD patients who scored lower than 7 on the MDQ at baseline, it is possible that our participants were particularly sensitive to the MDQ. Though the number was small, we did identify nine individuals who failed to meet the threshold of the MDQ at time 2, or the HCL‐32 at time 1 or time 2. Although we were unable to examine this statistically, these nine potential ‘false negative’ cases were nominally less likely to be white and under mental health services. This could indicate that these screening tools are ‘eurocentric’ (i.e., less valid in non‐white populations), who are also known to obtain poorer quality healthcare [34] or that those not under mental health services may have indeed had a less definitively bipolar illness.

### 4.3 Associations With Current Mood States and Clinical and Sociodemographic Factors

In line with previous research [18], the MDQ was positively associated with perceived conflict (ISS), as negative attitudes and hostile behaviours towards others can be associated with mania. Similarly, higher ISS activation scores, indicating an increased sense of behavioural and cognitive activation, as well as higher manic symptoms, were associated with both higher MDQ and HCL‐32 scores, and higher ISS global severity with higher HCL‐32 scores. Depressive symptoms were not associated with screening tool scores. The finding that participants with comorbid mental health diagnoses scored higher on the MDQ than those without was also expected, as comorbidities can have a negative impact on BD illness progression and symptom burden [35]. Somewhat more surprisingly, we found that ISS wellbeing, indicating psychological wellbeing, was associated with higher HCL‐32 scores, but a significant intercorrelation of this scale with both the ISS perceived conflict and the ISS activation scale [18] may explain this finding as indicative of some manic symptoms.

In line with findings of Hirschfeld [9], younger participants were more likely to receive higher screening tool scores. This may be because younger people can have poorer mood regulation abilities [36], resulting in heightened manic symptoms and therefore higher screening scores. We also found that fewer years since diagnosis were associated with higher HCL‐32 scores, which could be attributable to their likely younger age, and possibly shorter treatment and fewer illness management strategies. Similarly, fewer years since last hospitalisation were correlated with higher MDQ and HCL‐32 scores, possibly indicative of a more acute and less stable BD progression. Moreover, we found that BD‐I patients obtained higher scores than BD‐II and other BD participants on the HCL‐32, in line with the fact that BD‐I patients experience the most severe manic symptoms. We also found that (hypo)manic participants scored significantly higher on the MDQ than depressed or euthymic participants. This contrasts with the findings of Wang [37], who found no significant difference between MDQ scores across mood states. This is important as it may suggest that not only recall bias [14], but also current mood state could potentially influence MDQ scores. Interestingly, the same association was not found for the HCL‐32, and yet both scales are focused on symptoms of mania. We only found significant associations at time 1, and not at time 2. This may be due to participant attrition and resultant reduction in statistical power to find significant results at time 2.

### 4.4 Associations With Clinical Characteristics Changes

No clinical characteristic was found to be associated with screening score changes. This was surprising, as the included variables were associated with the screening scores cross‐sectionally.

### 4.5 Limitations and Implications

To the best of our knowledge, this is the first study directly comparing the longitudinal reliability and validity of the MDQ and HCL‐32 in a head‐to‐head fashion and with a relatively large sample size. However, these findings should be considered within the context of several limitations. Firstly, all participants had to meet the MDQ threshold of 7 at baseline to participate in the study, which may mean that we falsely excluded participants with a “true” BD diagnosis and that our sample was specifically sensitive to the screening tools. This could have resulted in higher screening tool scores than in the general BD population, inflating the agreement between screening scores and psychiatric diagnosis in our study and influencing the reliability calculations. Secondly, we did not recruit from a clinical setting or validate the accuracy of the diagnosis of our participants, resulting in the possibility that our participants may have been misdiagnosed as BD, which would have biased our results as well. Thirdly, we did not synthesise all available information from both screening tools, specifically BD duration, co‐occurrence of symptoms, and the associated impact, which differs between these measures. Future studies should consider this to maximise clinical utility. Fourthly, although we did examine the psychometric properties longitudinally, we only used a three‐month time span, which might not have been enough to capture changes over time. However, the test–retest reliability for the MDQ was already lower than expected within this relatively short time frame, suggestive of an even poorer reliability using longer durations. Furthermore, by examining the agreement between screening tool thresholds and formal diagnoses, we only assessed a small part of validity. Other validity types, e.g., agreement with other diagnostic methods, should be examined as well. Additionally, as expected in longitudinal studies, there was marked missing data, especially for the second time point. This may have biased our results, as study drop‐out is likely to be non‐random. Moreover, the study was conducted internationally, but we were unable to collect information on the participants' country of residence, which could have impacted the results. However, because the study was conducted in English and primarily advertised in the UK, Canada and the USA, it is unlikely that many non‐English speaking participants from other countries would have taken part. Lastly, the majority of our participants were female and white, which reduces the generalisability of our results to other ethnicities and genders.

Future studies should examine the psychometric properties of the MDQ and HCL‐32 in other cohorts, with more diverse, representative samples and over longer time periods. Furthermore, future studies with larger sample sizes should assess the features of people who fail to meet thresholds of screening tools and systematically examine possible reasons for false negative responses. Additionally, findings on factors associated with screening scores are inconclusive and future research should confirm those that we identified as well as examine other possible factors, such as family history, marital status, current employment status or the age of onset.

## 5 Conclusion

In conclusion, we found that the MDQ was not as reliable as previous studies suggested and performed poorer than the HCL‐32, both cross‐sectionally and longitudinally, in the specific patient sample, which largely consisted of white participants. Although the HCL‐32 may be recommended over the MDQ, both instruments should be used with caution, as they are designed for screening rather than diagnostic assessment. Moreover, when interpreting the results of the MDQ or HCL‐32, mood states and other demographic and clinical characteristics of the responders should be considered, as they may influence outcomes.

## Funding

Anna Tröger has received funding from the Deutsche Forschungsgemeinschaft (DFG, German Research Foundation) grant number GRK2773/1‐ 454245598.

## Conflicts of Interest

Thomas Richardson has been paid to deliver training on psychological therapies for Bipolar and receives royalties from a book on this topic. Allan H. Young reports a relationship with Flow Neuroscience that includes consulting or advisory and speaking and lecture fees. Allan H. Young reports a relationship with Novartis that includes consulting or advisory and speaking and lecture fees. Allan H. Young reports a relationship with Roche that includes consulting or advisory and speaking and lecture fees. Allan H. Young reports a relationship with Janssen Pharmaceuticals Inc. that includes consulting or advisory, funding grants, and speaking and lecture fees. Allan H. Young reports a relationship with Takeda that includes consulting or advisory and speaking and lecture fees. Allan H. Young reports a relationship with Noema Pharma that includes consulting or advisory and speaking and lecture fees. Allan H. Young reports a relationship with Compass that includes consulting or advisory, funding grants, and speaking and lecture fees. Allan H. Young reports a relationship with AstraZeneca that includes consulting or advisory and speaking and lecture fees. Allan H. Young reports a relationship with Boehringer Ingelheim that includes consulting or advisory and speaking and lecture fees. Allan H. Young reports a relationship with Eli Lilly that includes consulting or advisory and speaking and lecture fees. Allan H. Young reports a relationship with LivaNova that includes consulting or advisory, funding grants, and speaking and lecture fees. Allan H. Young reports a relationship with Lundbeck that includes consulting or advisory and speaking and lecture fees. Allan H. Young reports a relationship with Sunovion that includes consulting or advisory and speaking and lecture fees. Allan H. Young reports a relationship with Servier that includes consulting or advisory and speaking and lecture fees. Allan H. Young reports a relationship with Allegan that includes consulting or advisory and speaking and lecture fees. Allan H. Young reports a relationship with Bionomics that includes consulting or advisory and speaking and lecture fees. Allan H. Young reports a relationship with Sumitomo Dainippon Pharma that includes consulting or advisory services, and speaking and lecture fees. Allan H. Young reports a relationship with Sage that includes consulting or advisory and speaking and lecture fees. Allan H. Young reports a relationship with Neurocentrx that includes consulting or advisory and speaking and lecture fees. Rebecca Strawbridge reports a relationship with Janssen Pharmaceuticals Inc. that includes speaking and lecture fees. If there are other authors, they declare that they have no known competing financial interests or personal relationships that could have appeared to influence the work reported in this paper.

## Supporting information


**Supplementary S1** Clinical characteristics and mood states changes (paired samples *t*‐test/Chi‐Squared Test).

## Data Availability

The data that support the findings of this study are openly available in University of Southampton Institutional Repository at https://eprints.soton.ac.uk/468994/, reference number doi:https://doi.org/10.5258/SOTON/D2357.
