# Supplementary material for: Cross‐Sectional and Longitudinal Comparison of Commonly Used Screening Tools for Bipolar Disorders
Source: Bipolar Disord. 2026 Jun 21;28(5):e70133. doi: 10.1111/bdi.70133 (PMC13284449; doi:10.1111/bdi.70133)
Supplement: Supplementary file 1 — Supplementary S1 Clinical characteristics and mood states changes (paired samples t‐test/Chi‐Squared Test). [file BDI-28-0-s001.docx]

**Appendices**

**Supplement 1:** *Clinical characteristics and mood states changes (paired samples t-test/ Chi-Squared Test)*

| Questionnaire | *t^a^* | *χ2^b^* | p-value |
| --- | --- | --- | --- |
| ISS Global | -0.358 |  | 0.721 |
| ISS Activation | 0.486 |  | 0.627 |
| ISS Wellbeing | 0.194 |  | 0.846 |
| ISS Perceived conflict | 0.850 |  | 0.397 |
| ISS Depression index | 0.250 |  | 0.803 |
| ASRM | 0.164 |  | 0.870 |
| ReQoL | -1.030 |  | 0.305 |
| CESD | 0.573 |  | 0.568 |
| ISS Mood states |  | **61.854^***^** | <0.001 |

*Note:* ***p<0.001; significant results are highlighted in bold; a. Paired samples t-test for continuous variables (scores of questionnaires); b. Chi-Squared Test for polytomous variable (ISS mood states); ASRM: Altman Self-Rating Mania Scale; CESD: Center for Epidemiologic Studies Depression Scale; HCL-32: Hypomania symptom checklist-32; ISS: Internal State Scale, ISS Mood states (mania or hypomania/mixed state/euthymia/depression); MDQ: Mood Disorder Questionnaire; ReQoL: Recovering Quality of Life.
